# Disease Models & Mechanisms 2018: keeping you in the picture

**DOI:** 10.1242/dmm.034223

**Published:** 2018-02-01

**Authors:** Rachel Hackett

**Affiliations:** Disease Models & Mechanisms, The Company of Biologists, Bidder Building, Station Road, Histon, Cambridge, CB24 9LF, UK

With the advent of a new year, the Disease Models & Mechanisms (DMM) editorial team would like to share with you our reflections on the past year and our plans for the year ahead.

As a community journal, DMM is keen to support the next generation of biomedical scientists. During 2017, we launched ‘First person’ – a series of interviews with the first authors of a selection of papers published in DMM. We hope this helps early-career researchers promote themselves alongside their papers; you can see some of those we featured in our Interviews section. We've also launched an outstanding paper prize, to inspire young researchers. Starting with new submissions in 2018, DMM will award a prize ($1000) to the junior author(s) of the paper that is judged (by the journal's editors) to be the most outstanding publication in the journal that year. A short profile of the winner will be published in the journal. So, keep an eye out this time next year, when we will announce the inaugural winner of the prize.

As part of its financial support of early-career researchers, DMM has long offered Travelling Fellowships of up to £2500 to graduate students and postdoctoral researchers wishing to make collaborative visits to other laboratories. We've also been providing Conference Travel Grants since 2016. Following the huge success of this initiative, DMM will be doubling the funding available for this programme for 2018. These travel grants are aimed at early-career researchers wanting to attend scientific meetings, conferences, workshops and courses relating to the areas of research covered by the journal. Why not see whether you are eligible to apply?

As a community journal, DMM will continue to support conferences and workshops worldwide, in areas covered by the journal. In 2017, we supported the 8th Aquatic Animal Models of Human Disease Conference, Cancer Research UK Cambridge Institute 10th Anniversary Symposium, AACR Annual Conference, Bay Area Worm Meeting, Advances in Transgenic Animal Models and Techniques meeting, 24th Edition of the International Student Congress of (bio)Medical Sciences (ISCOMS), 15th Annual Meeting of the Complex Trait Community in collaboration with the Rat Genomics Community, Beatson Conference: Feeding the Beast, ZDM – 10th Annual Zebrafish Disease Models Conference, International Cell Competition Meeting (Japan), ISTT – 14th Transgenic Technology Meeting, EMBL Conference – Mammalian Genetics and Genomics, and the ‘A Revised Theory of Cancer’ symposium. We are still finalising our plans for 2018 – do get in touch if you think your meeting or symposium is an ideal fit for sponsorship from DMM.

DMM devotes time listening to its community, through surveys, social media, and at conferences and company workshops, as well as everyday interaction. We use what we've learned to then improve the journal, evaluating, amongst other aspects, its aims and scope, policies, processes, and the features and functionality we offer.

DMM aims to make life as easy as possible for our authors. To avoid lengthy submissions processes, authors can now submit a paper to DMM in any format (or transfer a paper directly from bioRxiv to DMM, without any need to reformat or re-enter information). We will only ask for what is absolutely necessary at submission. As part of this change, and recognising the importance of comprehensive Materials and Methods sections to aid transparency and reproducibility, we are also removing the Materials and Methods section from our length limit for an article. Importantly, the word limit itself will remain the same. The aim is to allow authors the space they need to describe their methods in sufficient detail for readers to fully understand and replicate the experiments conducted. We hope that this is making submission easier for all DMM authors, but it could have particular appeal for those authors who might have submitted their article elsewhere first – there is no need to reformat the article before submission to DMM. In addition, DMM will consider peer review reports from this previous submission to other journals. This new policy does mean that other requirements, such as file formats and sizes, our submission checklist, and provision of funding information, will move to the revision stage – at which point over 95% of papers will be accepted for publication.

Another factor that is important to our authors is speed from acceptance to publication. As part of this, DMM posts accepted author manuscripts soon after acceptance. And from issue 1 2018, DMM will be quicker to publish the final version, through the introduction of continuous publication. This ‘version of record’ will have been enhanced by expert in-house copyediting of text and figures, proofreading, typesetting and article layout, and conversion to printable PDF format and a fully linked full-text version. As soon as each article is ready, it will be immediately published online, rather than waiting for other articles in the issue to be completed. Authors and readers should primarily notice that the final version of the article is published more quickly, but behind the scenes, unpicking the implications of moving away from issue-based publishing has been a huge challenge.

Readers of DMM should certainly consider reviewing their DMM alert options, to ensure that they are receiving the alerts they need, when they need them. Alert preferences can be updated at http://dmm.biologists.org/alerts. Given the introduction of the General Data Protection Regulation (GDPR; if you haven't heard about this, you most certainly will soon) by the European Union (see http://www.biologists.com/what-is-gdpr/ for more details), we also encourage members of the DMM community to sign up for news alerts at http://www.biologists.com/subscribe/ to ensure they are kept up to date on other journal news, such as calls for papers, upcoming special issues, grant application deadlines and journal meetings.

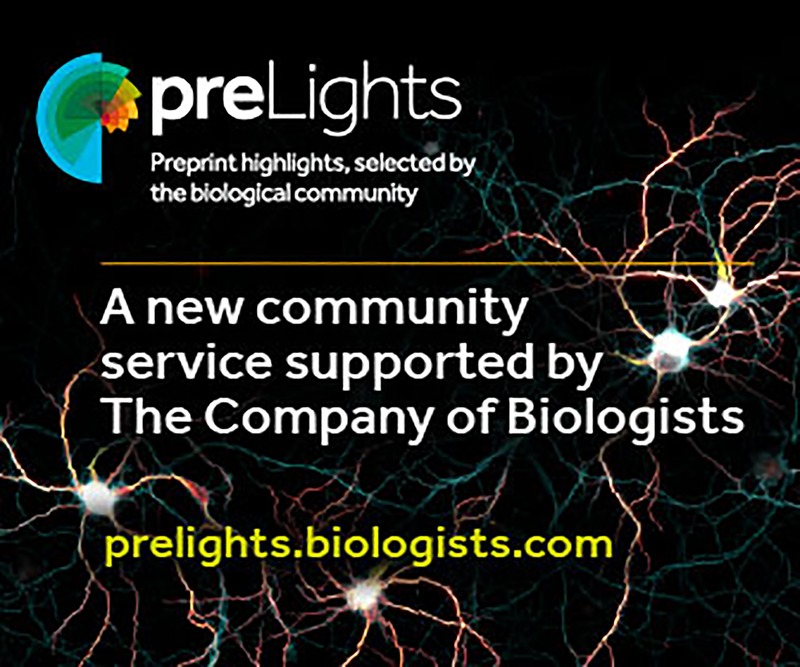

New for 2018: preLights – the new preprint highlights service run by the biological community and supported by The Company of Biologists. A team of scientists regularly review, highlight and comment on preprints they feel are of interest to DMM and the wider biological community.

You might also notice a new feature of the DMM web site – movies now ‘play in place’. If you are reading the full text of an article, you can click on a movie and rather than opening in a new window, the movie simply plays in that view. Movies are often vital to understanding a paper and this functionality makes it easier for readers to navigate articles.

In 2018, DMM plans to focus on the peer review process. Although DMM continually makes effort to hone and improve the experience of our peer reviewers, we strongly feel that more needs to be done, including the issue of reward and recognition. The Editors-in-Chief of all The Company of Biologists' journals met in February to begin discussions. In the meantime, DMM offers a huge thank you to all those who so generously give their time to review articles for DMM and who play such a fundamental role in the continuing success of DMM (see Supplementary Material for a full list of all those who completed a review for DMM in 2017).

## Supplementary Material

Supplementary information

