# Supplementary material for: Disease Models & Mechanisms 2018: keeping you in the picture
Source: Dis Model Mech. 2018 Feb 1;11(2):dmm034223. doi: 10.1242/dmm.034223 (PMC5894951; doi:10.1242/dmm.034223)
Supplement: Supplementary information [file dmm-11-034223-s1.pdf]

List of reviewers (1 January – 31 December 2017)

Noraishah Abdul-Aziz, University of Malaya, Malaysia
Seema Aceves, UCSD, USA
Usha Acharya, University of Massachusetts Medical School, USA
Ian Adcock, National Heart & Lung Institute, Imperial College London, UK
Dritan Agalliu, Columbia University Medical Center, USA
Imran Ahmad, The Beatson Institute for Cancer Research,, UK
Stephen Alexander, University of Missouri-Columbia, USA
Valérie Allamand, Institut de Myologie, France
Richard P Allen, Johns Hopkins University, USA
W. Ted Allison, University of Alberta, Canada
Maria Alves, Erasmus Medical Centre, The Netherlands
James Amatruda, UT Southwestern Medical Center, USA
Mahmood Amiry-Moghaddam, University of Oslo, Norway
James Amos-Landgraf, University of Missouri, USA
Robin Anderson, Peter MacCallum Cancer Centre, Australia
Samuel Aparicio, University of British Columbia/BCCA, Canada
Juan José Arredondo, Universidad Autónoma de Madrid, Spain
Ruben Artero, University of Valencia, Spain
Aboobaker Aziz, University of Oxford, UK
Istvan Baczko, University of Szeged, Hungary
Robert Baloh, Cedars-Sinai Regenerative Medicine Institute, USA
Thomas Baranski, Washington University, USA
Jeff Barclay, University of Liverpool, UK
Sami Barmada, University of Michigan, USA
Antonio Barrientos, Miller School of Medicine, University of Miami, USA
Joseph Bateman, King's College London, UK
Gillian Beamer, Tufts University, USA
Christine Beattie, Ohio State University, USA
Catherina Becker, University of Edinburgh, UK
Aaron Beedle, SUNY Binghamton University, USA
Ahmet Bekar, Uludag University, Turkey
Paola Bellosta, University of Trento, Italy
Heinz-Georg Belting, Universität Basel, Switzerland
Caterina Bendotti, Mario Negri Institute for Pharmacological Research, Milan, Italy
Johannes Berger, Medical University of Vienna, Austria
Andrew Berglund, University of Florida, USA
Charles Bevins, University of California, Davis, USA
Sanjay Bidichandani, University of Oklahoma, USA
Colin Bingle, The University of Sheffield Medical School, UK
Thomas Bird, University of Edinburgh, UK
Serge Birman, CNRS, ESPCI ParisTech, France
Trever Bivona, University of California San Francisco, USA
Helene Blasco, University of Tours, France
Anthony Bleyer, Wake Forest Baptist Medical Center, USA
Karen Blyth, Beatson Institute, UK
Bruce Bochner, Feinberg School of Medicine, Northwestern University, USA
Graeme Bolger, University of Alabama at Birmingham, USA
Maria Bondesson, University of Houston, USA
Nancy Bonini, University of Pennsylvania, USA
Joseph V. Bonventre, Brigham and Women's Hospital, Boston, USA
Stephanie Booth, University of Manitoba, Canada
Teresa Bowman, Albert Einstein College of Medicine, USA
Salvatore Bozzaro, University of Turin, Italy
Thomas Brand, Imperial College London, UK
Michael Brand, TU Dresden, Germany
Thomas Braulke, University Medical Center Hamburg-Eppendorf, Germany
Derrick Brazill, Hunter College, USA
Caroline Brennan, Queen Mary University of London, UK

Joe Bressler, Kennedy Krieger Institute, USA
Marco Brotto, University of Texas, Arlington, USA
Anthony Brown, Weill Cornell Medical College, USA
Valerie Brunton, University of Edinburgh, UK
Robert Bryson-Richardson, Monash University, Australia
Shawn Burgess, NHGRI/NIH, USA
Brant Burkhardt, University of South Florida, USA
Paul Burridge, Northwestern University, USA
Ed Burton, University of Pittsburgh, USA
Luca Businaro, CNR, Italy
Björn Busse, University Medical Centre Hamburg, Germany
Javier Caceres, University of Edinburgh, UK
Kim Caldwell, The University of Alabama, USA
Tito Calí, University of Padova, Italy
Laurent Calvier, UT Southwestern Medical Center, USA
Marina Campione, CNR Institute of Neurosciences, Italy
Francisco Campos, Health Research Institute of Santiago de Compostela (IDIS), Spain
Pere-Joan Cardona, Fundació Institut "Germans Trias i Pujol" (IGTP), Spain
S. Thomas Carmichael, David Geffen School of Medicine at UCLA, USA
Inaki Carril Mundinano, Monash University, Australia
Ana Luisa Carvalho, University of Coimbra, Portugal
Samit Chakrabarty, University of Leeds, UK
Ho Yin Edwin Chan, CUHK, China
Julia Charles, Brigham and Women's Hospital, USA
You-Ying Chau, University of Edinburgh, UK
Francisco Chavez, University of Chile, Chile
Yang-Mei Chen, The Second Affiliated Hospital of Chongqing Medical University, China
Yongchang Chen, Kunming University of Science and Technology, China
Zheng-Yi Chen, Massachusetts Eye & Ear Infirmary, Harvard Medical School, USA
Chia-Hsiung Cheng, Taipei Medical University, Taiwan
Cheng-Ting Chien, Academia Sinica, Taiwan
Seo-Kyung Chung, Swansea University, UK
Yuen-Li Chung, Institute of Cancer Research, UK
Erika Claud, University of Chicago, USA
Susanne Clee, University of British Columbia, Canada
Marc Clement, The University of North Carolina at Charlotte, USA
Don Cleveland, UCSD, USA
Hans Clevers, Hubrecht Institute, The Netherlands
Seth Coffelt, Glasgow University, UK
Don Cook, NIEHS, USA
Mark Cookson, NIH, USA
Jonathan Cooper, LA BioMed, Harbor-UCLA Medical Center, USA
Robert Cornell, University of Iowa, USA
Jorge Correia-Pinto, University of Minho, Portugal
Gino Cortopassi, UC Davis, USA
Susan Cotman, Massachusetts General Hospital, USA
Roger Cox, Medical Research Council, UK
Rachel Cox, Uniformed Services University, USA
Gage Crump, University of Southern California, USA
Emma Cunningham, Queen's University Belfast, UK
Christine Curcio, UAB School of Medicine, USA
Ales Cvekl, Albert Einstein College of Medicine, USA
Marija Cvetanovic, University of Minnesota, USA
James Dachtler, University of Durham, UK
Gokhan Dalgin, University of Chicago, USA
Christian Dani, CNRS, France
Davis Darryl, University of Ottawa Heart Institute, Canada
William Dauer, University of Michigan, USA
J. Muse Davis, University of Wisconsin, USA
Ivan de Curtis, San Raffaele Scientific Institute, Italy

Simone de Jong, King's College London, UK
Silvia De Rubeis, Icahn School of Medicine at Mount Sinai, USA
Adam Denes, University of Manchester, UK
Robert Desnik, Icahn School of Medicine at Mount Sinai, USA
Danelle Devenport, Princeton University, USA
David Dexter, Imperial College London, UK
Mary Dickinson, Baylor College of Medicine, USA
Tracey Dickson, Menzies Institute for Medical Research, University of Tasmania, Australia
Jeff Dilworth, University of Ottawa, Canada
Nicolas Diotel, Université de La Réunion, France
Gregory Dressler, University of Michigan, USA
Nicole Dubois, Icahn School of Medicine at Mount Sinai, USA
Carrie Duckworth, University of Liverpool, UK
Kirsten Sadler Edepli, NYU Abu Dhabi, United Arab Emirates
Dagmar Ehrnhoefer, BioMed X Innovation Center, Germany
Stephen Ekker, Mayo Clinic, USA
Marc Ekker, University of Ottawa, Canada
Matthew Ellis, Baylor College of Medicine, USA
Marina Emborg, University of Wisconsin-Madison, USA
Ruth Empson, University of Otago, New Zealand
Janice Endsley, University of Texas Medical Branch, USA
Charis Eng, Cleveland Clinic, USA
Kimberley Evason, Huntsman Cancer Institute, USA
Steven Farber, Carnegie Institution, USA
Colin Farquharson, Roslin Institute, UK
Olivia Farr, Beth Israel Deaconess Medical Center, Boston, USA
Mel Feany, Harvard Medical School, USA
Sarah-Maria Fendt, KU Leuven, VIB, USA
Yi Feng, University of Edinburgh, UK
Russell Ferland, Albany Medical College, USA
Laura Ferraiuolo, University of Sheffield, UK
Miguel Ferreira, Champalimaud Foundation, Portugal
Richard Festenstein, Imperial College London, UK
Bonnie Firestein, Rutgers, The State University of New Jersey, USA
Bernie Fischer, Duke University, USA
Elizabeth Fisher, University College London, UK
Flavia Fontanesi, University of Miami, USA
Roland Friedel, Icahn School of Medicine at Mount Sinai, USA
Denis Furling, Institut de Myologie, France
Francesca Fusco, Italian National Research Council, Italy
Michael Galko, University of Texas MD Anderson Cancer Center, USA
Nan Gao, Rutgers University, USA
Vidu Garg, Nationwide Children's Hospital, USA
Glòria Garrabou, Cellex, University of Barcelona, Spain
Philippe Gasque, University of La Reunion, France
Mathias Gautel, King's College London, UK
Daria Gavriouchkina, Weatherall Institute of Molecular Medicine, Oxford, UK
Silvia Gazzin, Italian Liver Foundation, Italy
Jadwiga Giebultowicz, Oregon State University, USA
John Gigg, University of Manchester, UK
Lenart Girandon, Educell Ltd., Slovenia
Daniel Gitai, Federal University of Alagoas, Brazil
Aaron Gitler, Stanford University School of Medicine, USA
Maurizio Giustetto, University of Turin, Italy
Inger Gjetsson, Goteborg University, Sweden
James Goldenring, Vanderbilt University School of Medicine, USA
Cayetano Gonzalez, IRB Barcelona, Spain
Pedro Gonzalez-Alegre, University of Pennsylvania, USA
Rose Goodchild, VIB/KU Leuven, Belgium
Martin Göpfert, University of Göttingen, Germany

Anand Goswami, Aachen University, Germany
Cheryl Gregory-Evans, University of British Columbia, Canada
Olli Grohn, University of Eastern Finland, Finland
Bernd Groner, Goethe-Universität Frankfurt am Main, Germany
Howard Gu, Ohio State University, USA
Carmen Guerra, CNIO, Madrid, Spain
Jorge Guerra Varela, Universidade de Santiago de Compostela, Spain
Shermali Gunawardena, SUNY at Buffalo, USA
Volker Haase, Vanderbilt University School of Medicine, USA
David Hackam, Johns Hopkins Children's Center, USA
Gabriel Haddad, University of California, San Diego, USA
Maria Hadjifrangiskou, Vanderbilt University, USA
Dieter Haffner, Hannover Medical School, Germany
David Hains, Indiana University School of Medicine, USA
Edward Hall, University of Kentucky, USA
Chris Hall, University of Auckland, New Zealand
Xianlin Han, Sanford Burnham Prebys Medical Discovery Institute, USA
Hideaki Hara, Gifu Pharmaceutical University, Japan
John Hardy, UCL Institute of Neurology, UK
Natasha Harvey, University of South Australia, Australia
Kieran Harvey, University of Melbourne, Australia
Cheryl Hawkes, Open University, UK
Jesse Hay, University of Montana, USA
David Hay, MRC Centre for Regenerative Medicine, UK
Tarik Haydar, Boston University School of Medicine, USA
Joan Heath, Walter and Eliza Hall Institute of Medical Research, Australia
Nicholas Heaton, Duke University School of Medicine, USA
Andreas Heinz, Charité—Universitätsmedizin Berlin, Germany
Clarissa Henry, University of Maine, USA
Yann Herault, IGBMC-ICS, France
Ann Hermansson, Lund University, Sweden
Héctor Herranz, University of Copenhagen, Denmark
Dan Hesselson, Garvan Institute of Medical Research, Australia
Michal Hetman, University of Louisville, USA
Ahlke Heydemann, University of Illinois at Chicago, USA
Herbert Hildebrandt, Medizinische Hochschule Hannover, Germany
Bob Hill, University of Edinburgh, UK
Susumu Hirabayashi, MRC Clinical Sciences Centre, Imperial College London, UK
Robert Hoffman, University of California San Diego, USA
Peter Hohenstein, The Roslin Institute, UK
Jorg Hohfeld, University of Bonn, Germany
Gregory Holmes, Mount Sinai School of Medicine Icahn Medical Institute, USA
Charles Hong, Vanderbilt University, USA
Julia Horsfield, University of Otago, New Zealand
Keith Hruska, Washington University School of Medicine in St Louis, USA
Wei Hsu, University of Rochester Medical Center, USA
Robert Huber, Trent University, Canada
Dirk Hubmacher, Icahn School of Medicine at Mount Sinai, USA
Toby Hurd, IGMM, University of Edinburgh, UK
Birgit Hutter-Paier, QPS Austria GmbH, Austria
El Chérif Ibrahim, Aix-Marseille Université, France
Robert Insall, University of Glasgow, UK
Mary Iovine, Lehigh University, USA
Adrian Isaacs, UCL Institute of Neurology, UK
Clare Isacke, Breakthrough Breast Cancer Research Centre, UK
Angelo Iulianella, Dalhousie University, Canada
Krzysztof Jagla, INSERM, Faculté de Médecine de Clermont-Ferrand, France
Cecilia Jimenez-Mallebrera, Hospital Sant Joan de Deu, Barcelona, Spain
Tian Jin, NIAID, NIH, USA
Jill Johnson, Aston University, UK

Arne Lund Jørgensen, Aarhus University, Denmark
Jean-Pierre Julien, Laval University, Canada
Madhuri Kango-Singh, University of Dayton, USA
Mike Karl, University of Washington, USA
Ravi Karra, Duke University School of Medicine, USA
Arthur Kaser, Cambridge University, UK
Koichi Kawakami, National Institute of Genetics, Japan
Robert Kelly, Institut de Biologie du Developpement de Marseille, France
Robert Kerbel, Sunnybrook Research Institute, Canada
Maurice Kernan, Stony Brook University, USA
Margaret Kielian, Albert Einstein College of Medicine, USA
Kazu Kikuchi, Victor Chang Cardiac Research Institute, Australia
Justin Kim, Duke University, USA
Tarja Kinnunen, University of Huddersfield, UK
Lynn Kirby, Temple University School of Medicine, USA
Janine Kirby, University of Sheffield, UK
Andreas Kispert, Medizinische Hochschule Hannover, Germany
Endre Kiss-Toth, University of Sheffield, UK
Arnaud Klein, Institut de Myologie, France
Jan Klohs, University of Zurich, Switzerland
Walter Kolch, University College Dublin, Ireland
Reinhard Koester, TU Braunschweig, Germany
Sulev Koks, University of Tartu, Estonia
Dennis Kolson, University of Pennsylvania, USA
Arjan Kortholt, University of Groningen, Germany
Peter Kotanko, Renal Research Institute, USA
Eva-Maria Krämer-Albers, Johannes Gutenberg University Mainz, Germany
Christof Kramm, University Medical Center, Gottingen, Germany
Doris Kretzschmar, Oregon Health and Science University, USA
Harm Krugers, University of Amsterdam, The Netherlands
Thomas Kukar, Emory University, USA
Satu Kuure, University of Helsinki, Finland
Jessica Kwok, University of Leeds, UK
Laura Lambert University of Alabama at Birmingham, USA
Thomas Langmann, University of Cologne, Germany
Stella Lanni, Hospital for Sick Children, Toronto, Canada
Lionel Larue, Institut Curie, France
Giovanna Lattanzi, CNR-Institute of Molecular Genetics, Italy
Nathan Lawson, University of Massachusetts Medical School, USA
Jun Hee Lee, University of Michigan, USA
Ernst Lengyel, University of Chicago, USA
Stewart Levine, NHLBI, NIH, USA
Mark Lewandoski, NCI, USA
Yuqing Li, University of Florida, USA
Graham Lieschke, Australian Regenerative Medicine Institute, Australia
Xi Lin, Emory University School of Medicine, USA
Corinne Linardic, Duke University Medical Center, USA
Troy Littleton, Baylor College, USA
Shu Fang Liu, The Feinstein Institute for Medical Research, Hofstra/Northwell School of Medicine, USA
Alberto Lleo, Research Institute of the Hospital de la Santa Creu i Sant Pau, Spain
Miguel López, University of Santiago de Compostela, Spain
Christian Lorson, University of Missouri, USA
Erik Lundquist, University of Kansas, USA
Livio Luongo, Università degli Studi della Campania, Italy
Georges Lutfalla, Université Montpellier, France
David Lynch, University of Pennsylvania, USA
Liset M. de la Prida, Instituto Cajal, Spain
Xianjue Ma, Yale School of Medicine, USA
Nanna MacAulay, University of Copenhagen, Denmark

Paolo Macchi, University of Trento, Italy
Patricia Maciel, University of Minho, Portugal
Donna Maglott, NCBI, USA
Jocelyne Magre, INSERM, France
Richard Mains, University of Connecticut Health Center, USA
Taija Mäkinen, Uppsala University, Sweden
Silvia Mandillo, CNR-Istituto di Biologia Cellulare e Neurobiologia, Italy
Mao, University of California, San Francisco, USA
Alain Martelli, Pfizer Rare Disease Research Unit, USA
Paul Martin, University of Bristol, UK
Rudolf Martini, University Hospital Würzburg, Germany
Barry McColl, University of Edinburgh, UK
Larry McDaniel, The University of Mississippi Medical Center, USA
Annemarie Meijer, Leiden University, The Netherlands
Giampaolo Merlini, University of Pavia, Italy
Greg Mertz, University of New Mexico, USA
Fabrizio Michetti, Universita Cattolica del S. Cuore, Italy
Marco Milan, IRB Barcelona, Spain
James Minchin, University of Edinburgh, UK
Helena Minye, David Geffen School of Medicine, University of California, USA
Marina Mione, University of Trento, Italy
Christopher Mirchell, University of Ulster, UK
Masayuki Miura, University of Tokyo, Japan
Ken Moberg, Emory University, USA
Gabriela Morali, Unidad de Investigación Médica en Farmacología, Mexico
Paula Moran, University of Nottingham, UK
Margaret Morris, University of South Wales, Australia
Richard Mort, Lancaster University, UK
Jen Morton, The Beatson Institute, UK
Rory Morty, Max Planck Institute for Heart and Lung Research, Germany
Amrit Mudher, University of Southampton, UK
Keith Murphy, UCD Conway Institute of Biomolecular and Biomedical Research, Ireland
Erik Musiek, Washington University of St Louis, USA
Laura Musselman, Binghamton University, USA
Steven Mutsaers, University of Western Australia, Australia
Indira Mysorekar, Washington University School of Medicine, USA
Shanmugam Nagarajan, University of Pittsburgh, USA
Anneline Nansen, Zealand Pharma, Denmark
Marek Napierala, University of Alabama at Birmingham, USA
Mihai Netea, Radboud Institute for Molecular Life Sciences, The Netherlands
Stephan Neuhauss, University of Zurich, Switzerland
Jeffrey Neul, Baylor College of Medicine, USA
Philipp Niethammer, Memorial Sloan-Kettering Cancer Center, USA
Angela Nieto, Consejo Superior de Investigaciones Científicas, Universidad Miguel Hernández, Spain
Nikolay Ninov, CRTD, Dresden, Germany
Will Norton, University of Leicester, UK
Bennett Novitch, UCLA School of Medicine, USA
Niamh Nowlan, ICL, UK
Lauryl Nutter, The Centre for Phenogenomics, Canada
Judith Ogilvie, Saint Louis University, USA
Kevin Ohlemiller, Washington University School of Medicine, St Louis, USA
Harry Orr, University of Minnesota, USA
Michael Pack, University of Pennsylvania, USA
Antonio Pagán, Cambridge University, UK
Gholam Pajenda, Medical University Vienna, Austria
Victoria Palau, East Tennessee State University, USA
Michael Palladino, University of Pittsburgh, USA
Leo Pallanck, Washington University School of Medicine, USA
David Parichy, University of Washington, USA
Sung Wook Park, Seoul National University College of Medicine, Republic of Korea

Clarissa Parker, Middlebury College, USA
Alex Parker, CRCHUM, Université de Montréal, Canada
Laura Parkkinen, Oxford University, UK
Marie-Laure Parmentier, IGF, France
Michael Parsons, Johns Hopkins University, USA
Linda Partridge, UCL, UK
Annalisa Pastore, Kings College London, UK
Margaret Pearce, University of the Sciences, USA
Chris Peers, University of Leeds, UK
Diego Perez-Tilve, University of Cincinnati, USA
Muriel Perron, Université Paris Sud, France
Nikolaj Petersen, Orphazyme, Denmark
Patrice Petit, Paris Descartes University, France
Toby Phesse, Cardiff University, UK
Andrew Pitsillides, Royal Veterinary College, UK
Anna Planas, Institut d' Investigacions Biomèdiques de Barcelona (IIBB), Spain
Andrea Pollard, Imperial College London, UK
Steve Pollard, University of Edinburgh, UK
Mark Pook, Brunel University London, UK
David Porteous, IGMM, UK
David Pritchard, University of Liverpool, UK
Catrin Pritchard, University of Leicester, UK
Hélène Puccio, Institut de Génétique et de Biologie Moléculaire et Cellulaire (IGBMC), France
Christine Radtke, Vienna General Hospital, Austria
Raj Rajakumar, University of Western Ontario, Canada
Sepand Rastegar, Karlsruhe Institute of Technology, Germany
Angel Raya, CMRB, Spain
Roger Reeves, Johns Hopkins University School of Medicine, USA
R. Lee Reinhardt, National Jewish Health, USA
Krzysztof Reiss, LSU Health, New Orleans, USA
Simone Renner, Ludwig-Maximilians-Universität München, Germany
Stephen Renshaw, University of Sheffield, UK
Jason Rihel, UCL, UK
Ryan Roberts, Nationwide Children's Hospital, USA
Randall Roper, Indiana University Purdue University Indianapolis, USA
Norman Rosenblum, University of Toronto, Canada
Leonardo Rossi, University of Pisa, Italy
Florence Ruggiero, Université Lyon, France
David Sacks, NAIAD, USA
Erik Sahai, The Francis Crick Institute, UK
Lynn Sakai, Oregon Health & Science University and Shriners Hospital for Children, USA
Pedro Salas, University of Miami, USA
Marco Sandri, Venetian Institute of Molecular Medicine, Italy
Filippo Santorelli, IRCCS, Italy
Marco Sardiello, Baylor College of Medicine, USA
Smita Saxena, University of Bern, Switzerland
Manfred Schartl, Biocenter of the University of Wurzburg, Germany
Amnon Schlegel, University of Utah School of Medicine, USA
Katharina Schmitt, German Heart Institute, Germany
Frank Schnorrer, Institut de Biologie du Développement de Marseille, France
Paul Schofield, University of Cambridge, UK
Laurence Schook, University of Illinois, USA
Tony Schountz, Colorado State University, USA
Ulrich Schraermeyer, University of Tübingen, Germany
Michael Schuliga, University of Newcastle, Australia
Michael Schumacher, University of Paris-Sud, France
Daryl Scott, Baylor College of Medicine, USA
Florenci Serras, Universitat de Barcelona, Spain
Carolyn Sevier, Cornell University College of Veterinary Medicine, USA
Nutan Sharma, Massachusetts General Hospital, USA

Toshio Shibata, Kyusyu University, Japan
Ramesh A. Shivdasani, Dana-Farber Cancer Institute, USA
James Shorter, University of Pennsylvania School of Medicine, USA
Veronica Shubayev, UCSD, USA
Florian Siebzenrubl, Cardiff University School of Biosciences, UK
Dirk Sieger, University of Edinburgh, UK
Debra Silver, Duke University School of Medicine, USA
Alex Simon, University of Arizona, USA
Debora Sinner, Cincinnati Children's Hospital Medical Center, USA
Michael Sittinger, Berlin-Brandenburg Center for Regenerative Therapies, Germany
Hazel Sive, Whitehead Institute, USA
Efthimios Skoulakis, BSRC "Alexander Fleming", Greece
John Sled, Hospital for Sick Children, Canada
William Sly, St Louis University School of Medicine, USA
Fahrida Sohrabji, Texas A&M Health Science Center, USA
Regina Sordi, Queen Mary University of London, UK
Raj Srivastava, A*STAR Institute, Singapore
Kryn Stankunas, University of Oregon, USA
Donald Stein, Emory University, USA
Jacqueline Stephens, Pennington Biomedical Research Center, USA
Scott Stewart, University of Oregon, USA
Peter Stirling, BC Cancer Agency, Canada
Rainer Straub, University Medical Centre Regensburg, Germany
Hélène Strick-Marchand, Institut Pasteur, France
Elinor Sullivan, OHSU, USA
David Suter, Ecole Polytechnique Fédérale de Lausanne, Switzerland
Masatoshi Suzuki, University of Wisconsin, USA
Kathy Svoboda, Texas A&M Dentistry, USA
Boris Tabakoff, University of Colorado School of Pharmacy, USA
Jacqueline Tabler, Max Planck Institute of Molecular Cell Biology and Genetics, Germany
Jordi Tamarit, Lleda University, Spain
Ernst Tamm, University of Regensburg, Germany
Filippo Tempia, University of Torino, Italy
Gaetano Thiene, University of Padova, Italy
Tim Thomas, Walter and Eliza Hall Institute of Medical Research, Australia
Glen Tibbits, Simon Fraser University, Canada
Ignacio Torres, Aleman Cajal Institute, Spain
David Tosh, University of Bath, UK
Dan Tracey, Indiana University Bloomington, USA
Paul Trainor, Stowers Institute for Medical Research, USA
Andreas Traweger, Paracelsus Medical University, Austria
Jacques Tremblay, Universitaire de Québec, Canada
Li-Huei Tsai, MIT, USA
Maria Tsoli, Children's Cancer Institute Australia, Australia
Elizabeth Tucker, Johns Hopkins Hospital, USA
Claire Turner, University of Sheffield, UK
Benjamin Tycko, Columbia University Medical Center, USA
Asier Unciti-Broceta, University of Edinburgh, UK
Renée Van Amerongen, University of Amsterdam, The Netherlands
Jeremy Van Raamsdonk, Van Andel Research Institute, USA
Kartik Venkatachalam, The University of Texas Health Science Center at Houston, USA
Dietmar Vestweber, Max-Planck-Institute for Molecular Biomedicine, Germany
Mark Vickers, University of Auckland, New Zealand
Alex Vieira, University of Pittsburgh, USA
Jenny A. Visser, Erasmus MC, The Netherlands
Richard Wade-Martins, University of Oxford, UK
Christian Waeber, University College Cork, Ireland
Kathryn Wagner, Johns Hopkins University, USA
Satoshi Wakisaka, Osaka University Graduate School of Dentistry, Japan
Tao Wang, National Institute of Biological Sciences, China

Yanan Wang, Leiden University Medical Center, The Netherlands
Liewei Wang, Mayo Clinic, USA
Alastair Watson, UEA, UK
Heming Wei, National Heart Centre Singapore, Singapore
Brant Weinstein, NICHD, USA
Cheryl Wellington, University of British Columbia, Canada
Rong Wen, University of Miami, USA
Robert Wheeler, University of Maine, USA
Robin Williams, Royal Holloway, UK
Luke Wiseman, Scripps Research Institute, USA
Andrew Wood, IGMM, UK
Jonathan Wood, University of Sheffield, UK
Ian Wood, University of Leeds, UK
Alison Woollard, University of Oxford, UK
Anna Wredenberg, Karolinska Institute, Sweden
Mingming Wu, Cornell University, USA
Wolfgang Wurst, Helmholtz Zentrum Munich, Germany
Ying Xia, University of Cincinnati, USA
Xiaolei Xu, Mayo Clinic, USA
Chunhui Xu, Emory University School of Medicine, USA
Shinya Yamamoto, Baylor College of Medicine, USA
Bing Ye, University of Michigan, USA
Yvette Yien, Brigham and Women's Hospital, USA
Mark Yorek, University of Iowa, USA
H. Joseph Yost, University of Utah, USA
Ihor Yosypiv, Tulane University School of Medicine, USA
Guillermo Zalba, Universidad de Navarra, Spain
Erica Zamberletti, University of Insubria, Italy
Daniela Zarnescu, University of Arizona, USA
Andrew Zelhof, Indiana University, USA
Sheng Zhang, University of Texas, USA
Kang Zhang, Veterans Administration Healthcare System, USA
Lei Zhao, Shandong Provincial Cancer Hospital and Institute, China
Gong Zhiyuan, National University of Singapore, Singapore
Berislav Zlokovic, Keck School of Medicine of USC, USA
Robert Zweigerdt, Hannover Medical School, Germany
